# A Novel Environmental Justice Indicator for Managing Local Air Pollution

**DOI:** 10.3390/ijerph15061260

**Published:** 2018-06-14

**Authors:** Jing Zhao, Laura Gladson, Kevin Cromar

**Affiliations:** Marron Institute of Urban Management, New York University, 60 5th Avenue, New York, NY 10011, USA; jz2181@nyu.edu (J.Z.); laura.gladson@nyu.edu (L.G.)

**Keywords:** environmental justice, outdoor air pollution, fine particulate matter, job accessibility, mobility index, urban management

## Abstract

Environmental justice efforts in the United States seek to provide equal protection from environmental hazards, such as air pollution, to all groups, particularly among traditionally disadvantaged populations. To accomplish this objective, the U.S. EPA has previously required states to use an environmental justice screening tool as part of air quality planning decision-making. The generally utilized approach to assess potential areas of environmental justice concern relies on static comparisons of environmental and demographic information to identify areas where minority and low income populations experience elevated environmental exposures, but does not include any additional information that may inform the trade-offs that sub-populations of varying socio-demographic groups make when choosing where to reside in cities. In order to address this limitation, job accessibility (measured by a mobility index defining the number of jobs available within a set commuting time) was developed as a novel environmental justice indicator of environmental justice priority areas at the local level. This approach is modeled using real-world data in Allegheny County, PA (USA), and identifies areas with relatively high levels of outdoor air pollution and low access to jobs. While traditional tools tend to flag the poorest neighborhoods for environmental justice concerns, this new method offers a more refined analysis, targeting populations suffering from the highest environmental burden without the associated benefits of urban living.

## 1. Introduction

National efforts to improve air quality have seen great success in the United States since their mid-twentieth century inception. Yet these and other environmental health improvements vary locally, often neglecting disadvantaged urban populations such as minority groups and low-income populations who have traditionally had little political sway over environmental policy decisions impacting their communities [1]. Recognition of this inequity led to the development of a new national endeavor by the early 1990s—termed *environmental justice—*which sought to provide equal protection to all groups from environmental hazards [2]. The definition of environmental justice has evolved since its inception, which now includes an added focus on informed individual choice and empowerment in public decision-making that is as equally important as the direct measurements of environmental conditions.

Historically, minorities and other susceptible populations have suffered the greatest environmental inequities, and recent academic research consistently reveals persisting disparities in exposure to environmental harms by class and race. The UCC’s Commission for Racial Justice report, “Toxic Waste and Race at Twenty”, states that minority groups continue bearing the greatest health burdens from hazardous waste produced in the U.S. [3]. Additionally, a decade-long national study revealed that while average toxin exposures and their associated health risks are falling among the general population, exposure and risk among African Americans has remained the same [4]. Some progress has been made, however, as revealed in the EPA’s 2017 Environmental Justice Progress Report, which found that 92% of low-income individuals live in areas which presently meet national PM_2.5_ standards. However, the remaining 8% represent those with the greatest economic and health disparities, making environmental justice issues especially difficult to address. These at-risk individuals total to over 4 million, and primarily reside in cities [5].

Consideration of environmental justice issues is a required step for some aspects of permitting, enforcement, and compliance of air quality rules in the United States [6]. The U.S. Environmental Protection Agency (EPA) has created a tool for making these assessments, called EJSCREEN, which can be used as a preliminary step when considering environmental justice issues (www.epa.gov/ejscreen). Beyond its required use by the EPA, EJSCREEN is widely employed by various other government, industry, and community groups [5]. This online application provides a rough overlay of environmental health and demographic indicators across any specified region, allowing federal agencies and local municipalities alike to assess what areas within a jurisdiction may suffer from the greatest environmental inequities. For example, EJSCREEN can provide an output for a fine particulate matter (PM_2.5_) index by multiplying three variables: the environmental indicator (calculated from annual air pollution data), the demographic index (determined by comparing local averages of susceptible populations to national statistics), and the population count of each census block group [7]. Using this information, policymakers can determine what additional analysis, outreach, or programs may be needed in order to address air pollution impacts in the identified locations.

The identification of the poorest areas within a city that have elevated pollution levels, using tools such as EJSCREEN, represents a traditional approach to environmental justice, but may not reflect the choices and trade-offs that individuals make when choosing a location of residence. For example, air quality is generally worse in cities compared to less populated areas, where the high density of low-income and minority populations has the potential to inflate environmental inequities [8]. However, despite the potential negative trade-offs of urban living (e.g., increased housing costs, crowding, and increased environmental risks), the rate of population growth is highest in cities compared to areas with lower levels of population density [9].

The primary purpose and advantage of cities is to function as an abundant labor market, which drives large populations to urban areas despite potentially negative tradeoffs such as increased air pollution [10]. Studies have shown that the economic outputs of labor markets increase with market size, explaining the continuing growth of large cities despite the drawbacks to urban living [11]. Additionally, not only do higher urban densities of businesses and industries offer a greater raw number of jobs to prospective workers, but provide higher wages for urban workers due in part to more efficient and varied organizational structures [12]. It is therefore important to consider job accessibility when evaluating the equity conditions of urban dwellers.

With this in mind, the present study reports on the development of a novel indicator of environmental justice for air pollution by directly weighing the primary trade-offs that are made based on the geographic location of urban and suburban residents. This approach uses access to jobs, measured by a mobility index defining the number of jobs available within a specified commuting time, as a way to identify environmental justice priority areas at the local level. In essence, this approach seeks to identify locations that shoulder the negative aspects of living in close proximity to an urban center (elevated levels of outdoor air pollution) without experiencing the primary benefits of major cities (increased access to jobs). Incorporating these primary trade-offs of urban living, both positive and negative, may improve the effectiveness of environmental justice indicators in identifying areas with disproportionate air quality burdens within a jurisdiction.

## 2. Materials and Methods

Allegheny County in Pennsylvania, USA was selected for analysis due to its relatively high levels of outdoor air pollution and strong local economy. The county seat in Pittsburgh was historically a key steel manufacturing area, but is now home to several major companies with abundant employment opportunities. The remaining coke and power plants, plus city traffic emissions, contribute the county’s local air pollution problem [13]. In fact, Allegheny was one of only 13 counties nationwide to be designated by the EPA as a nonattainment area for the 2012 annual PM_2.5_ standards [14]. Given the attractiveness of this city as a labor market and the environmental burden that its residents endure, it is an ideal location for illustrating how environmental justice might be reconsidered as part of ongoing air quality management planning.

**Figure 1:**
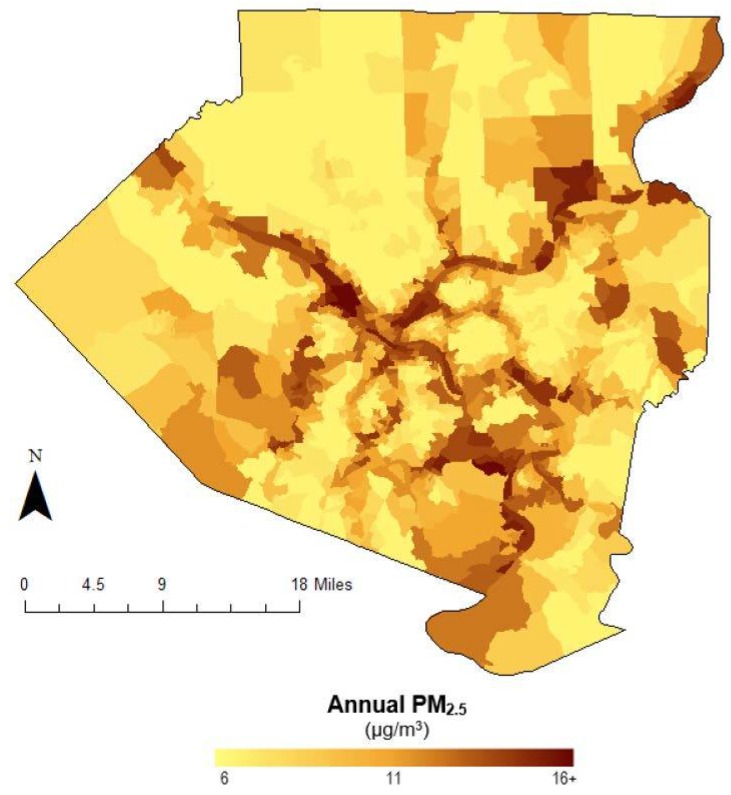
Annual PM_2.5_ concentrations by census block group in Allegheny County, PA. Values estimated using land-use regression of monitors deployed at 36 locations during 2013–2014.

PM_2.5_ estimates for Allegheny County were acquired from research conducted at Carnegie Mellon University (CMU) [15]. PM_2.5_ measurements were gathered in the summer (August) of 2013 and winter (December to January) of 2013–2014 from 36 monitor sites, and annual levels across the county were determined using a Land Use Regression (LUR) model as developed in the ESCAPE project [16]. The spatial distribution of annual PM_2.5_ concentrations is shown in Figure 1.

Using OpenTripPlanner (OTP) [17], job accessibility was estimated as the number of job opportunities available within a 45 min commute from a home location by public transit, biking, and/or walking. The key elements of a job accessibility calculation are the spatial distribution of jobs, the accessibility provided by the transportation system, and the time and cost threshold [18,19,20]. The fare for the public transportation system in Allegheny County is $2.75 per ride, which is reasonable and invariable to travel distance; as such, the job accessibility calculation in this study was calculated based on the first three elements without a cost threshold. Based on discussion in Access Across America: Transit 2015 [21], the average walking speed was set to 5 km per hour (3.1 miles per hour), and the maximum daily walking distance to 0.804 km (0.5 miles). The average biking speed was set to 19.3 km per hour (12 miles per hour).

High Performance Computing (HPC) at New York University was used to run Python URLs [22] to request OTP Analyst server data, generating transit scopes for all census blocks in Allegheny County based on transport network maps from OpenStreetMap (OSM) and General Transit Feed Specification (GTFS). The mobility index, which estimates the number of jobs accessible within a 45 min commute using public transportation, was determined by intersecting the scope isochrone maps for each home location with census-determined work locations. For each home location, a GeoJSON map was pulled from OpenTripPlanner containing the scope and all the coordinates of work locations that each home address can access within 45 min using public transportation. This was then processed using the GeoPanda Python package as an isochrone map and intersected with a job distribution map of Allegheny County, pulled from the web-based application *OnTheMap* including all jobs based on 2015 U.S. Census data [23], to get a map of job locations located within the 45 min scope. By using Python, the total number of jobs accessible for one home address was calculated by summing up the number of jobs available in each work location within the 45 min scope. The results of intersection were then compiled into a single dataset that contained job accessibility and geographic information of all census blocks. Job accessibility for three different weekday time periods (8:00 a.m., 12:00 p.m. and 4:00 p.m.) was generated. These three datasets were then averaged to get daily job accessibility, and aggregated to the census block group level. After going through the same method for every home location, a distribution map of the mobility index was generated through ArcGIS (Esri, Redlands, CA, USA) by using the coordinates of the home locations and their corresponding mobility index.

The identification of environmental justice priority areas was determined at the block group level based on three criteria: annual PM_2.5_ concentrations from the land-use regression model, income statistics from the 2012 to 2016 American Community Survey (ACS) 5-Year Estimates from the United States Census Bureau [24], and job accessibility data estimated using the previously described mobility index. Areas were identified that had: annual PM_2.5_ levels greater than 12 µg/m^3^, corresponding to the U.S. national ambient air quality standard; annual median household levels below $54,357 (the county average); and access to less than 50,000 (primary cut-point) or 100,000 (secondary cut-point) jobs accessible within a 45 min public transit, biking, and/or walking commute. Census block groups meeting all three criteria above were identified in this analysis as environmental justice priority areas.

Areas identified in the current study as environmental justice priority areas were compared to block groups identified using EJSCREEN. The raw database for Allegheny County was accessed through the EJSCREEN website (https://www.epa.gov/ejscreen/download-ejscreen-data), which is provided at the block group level. The environmental index for particulate matter was selected as the variable for identifying areas of high environmental justice concerns, labeled “Percentile PM_2.5_ Level in Air—Primary EJ Index based on Primary 2-factor Demographics” (field name P_PM25_D2) in EJSCREEN datasets. The particulate matter index for each block group is calculated by multiplying three variables together: the PM_2.5_ levels in air (in µg/m^3^ based on 2013 annual averages), the difference between the demographic index of the block group and the national demographic index, and the block group’s population count. The demographic index is defined as the average of the percent low income and percent minority populations in the area of interest. Primary and secondary cut-points for traditional priority areas were set at EJSCREEN particulate matter index values of 95th percentile and above and between the 90th and 95th percentiles, respectively, based on standard cut-offs presented in EJSCREEN reports [7]. These traditional analysis results were then compared with block groups identified using the new method of the present analysis.

It has been recognized that U.S. Census ACS 5-Year Estimates, particularly the median household income variable, have potential reliability problems [25]. However, the decision to use this data source for demographic variables in the present analysis was made in order to increase the comparability between traditional and current study methods, since these estimates are the source of all demographic data used in EPA EJSCREEN index calculations [7]. In addition, income has been aggregated into “below” and “above” median household income levels following suggestions made by Folch et al. regarding how to best handle ACS income data [25].

Using all jobs in the analysis of job accessibility inherently includes skilled positions not available to all individuals, particularly those disadvantaged populations with lower education levels. However, there is good evidence that job growth in high-education sectors of the job market creates additional jobs in other sectors, termed the “local multiplier” effect [26]. In fact, a report by the Bay Area Council’s Economic Institute revealed that for every high-tech sector job, 4.3 additional jobs were created in the local goods and services sector in the study region. These values vary by sector and location, but the principle has held true in various regions across the U.S. [27,28]. In light of this, the analysis relied on all jobs to calculate the mobility index, with the acknowledgment that individuals are not qualified for every available position but allowing for the assumption that access to higher numbers of jobs is beneficial as compared to having access to lower numbers of jobs.

## 3. Results

**Figure 2:**
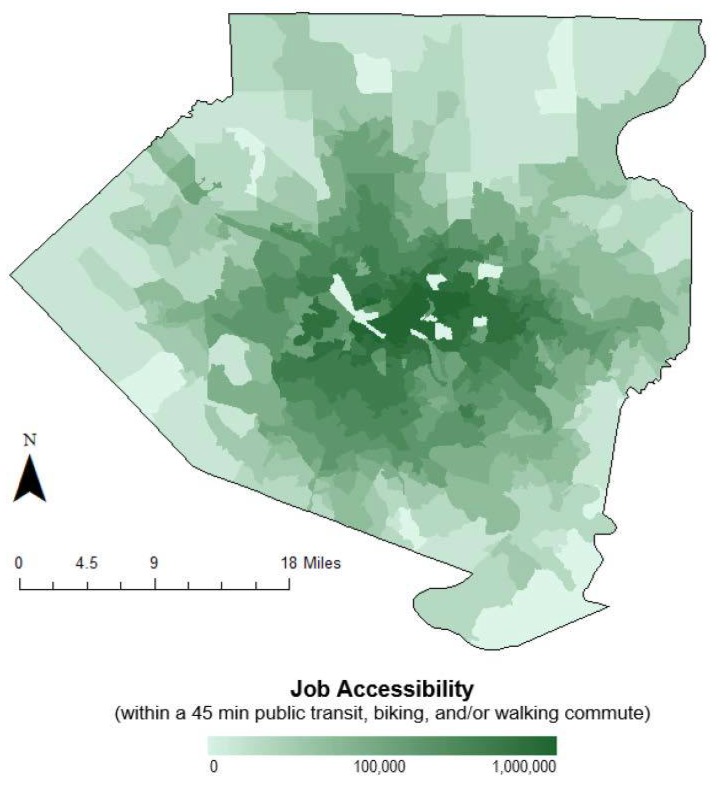
Log-adjusted number of jobs accessible by census block group in Allegheny County, PA. Job accessibility is estimated using publically available transportation and jobs data.

Air pollution data reveals a fair level of heterogeneity of pollution concentration across Allegheny County. Figure 1 presents average annual PM_2.5_ concentrations across all 1100 block groups. A comparison of landscape characterization information provided by Li et al. reveals that municipalities with PM_2.5_ levels above the NAAQS standard are highly correlated to the distribution of freeways, major rivers, and large emissions contributors, particularly coal combustion sources in the Ohio River Valley [15,29,30]. Job accessibility showed a more definitive pattern. Figure 2 depicts a log-adjusted spread of job accessibility by block group across the county. As expected, those living near the city center have the highest measured mobility indices due to both closer proximity to the highest numbers of jobs and better access to public transportation.

**Figure 3:**
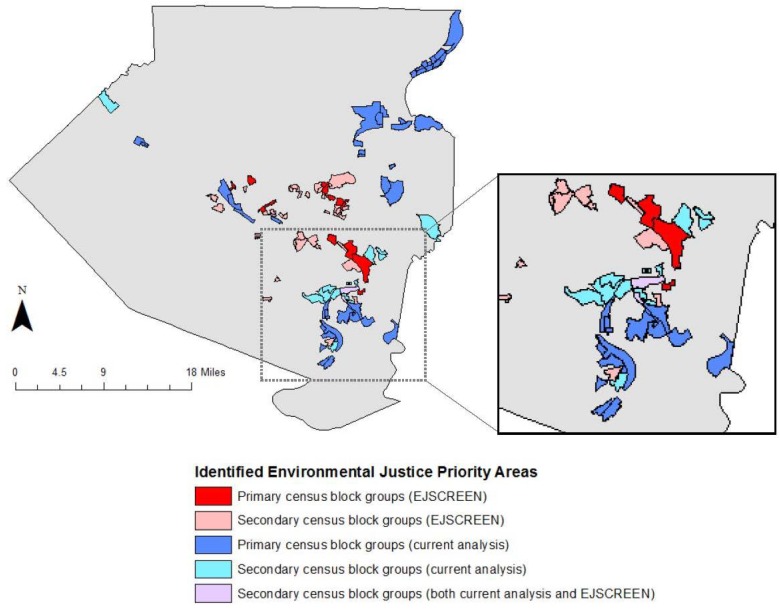
Environmental justice priority areas identified in Allegheny County, PA, by EJSCREEN and a newly developed environmental justice indicator that uses job accessibility as a parameter. Primary census block groups identified using EJSCREEN represent index values in the 95th percentile and above; secondary traditional priority areas are between the 90th and 95th percentile. Primary census block groups identified in the current analysis are those with PM_2.5_ concentrations >12 µg/m^3^ and income <$54,357, and access to less than 50,000 jobs; secondary block groups have access to between 50,000 and 100,000 jobs.

The map in Figure 3 compares block groups identified by traditional EJSCREEN analysis and those identified in the present study to merit additional consideration for environmental justice concern. “Primary” and “secondary” areas have been identified, corresponding to areas with the highest concern of disproportionate burden of air pollution. Primary areas of concern identified using traditional methods were defined as those with EJSCREEN index values at the 95th percentile and above, which the EPA defines as places where environmental justice efforts should be concentrated [5]. EJSCREEN index values between the 90th and 95th percentiles are also shown as secondary areas for traditional methods. The block groups identified by EJSCREEN tend to be concentrated around the city center, while those identified in this new analysis are located primarily in the northeast and southeast regions of the county (see detail for Figure 3). Only two census block groups, both secondary areas, overlap between the two methods.

Table 1 compares the characteristics of primary and secondary census block groups identified by EJSCREEN and the present analysis. These results indicate that the size of the affected populations living in block groups identified by EJSCREEN and in the current analysis is qualitatively comparable. However, population-weighted averages for pollution concentrations and jobs vary dramatically using the two different approaches. Outdoor concentrations of PM_2.5_ are much higher among block groups identified in the present study as compared to areas identified using EJSCREEN. From both a regulatory and health perspective, the difference in annual PM_2.5_ concentrations of 2.8 µg/m^3^ (11.0 µg/m^3^ for areas identified using EJSCREEN vs. 13.8 µg/m^3^ for areas identified in this analysis) is substantial.

The median incomes of individuals living in primary and secondary areas identified using EJSCREEN were slightly lower than the median incomes of individuals living in areas identified in the current analysis (see Table 1). These relatively lower income levels compared to the present analysis is not entirely unexpected due to the heavy weighting of income in the equations used in EJSCREEN.

Lastly, the number of jobs accessible in the areas identified in the current analysis is approximately one tenth of the number available in EJSCREEN-identified block groups. In fact, the primary areas identified as having environmental justice concerns using EJSCREEN were among the best in the county in regards to job accessibility. More detailed information of the individual census block groups that were identified by EJSCREEN and in the present analysis is shown in Tables S1–S4.

## 4. Discussion

The motivation for developing updated indicators of environmental justice is to better assist local air quality managers in identifying geographic areas within their jurisdiction that experience the most disproportionate impacts of outdoor air pollution. While it may not be immediately intuitive, the most critical areas to focus on are not necessarily locations with the absolute highest levels of outdoor air pollution. As an example, the Upper East Side in New York City has some of the highest levels of ambient air pollution in the city, but as one of wealthiest neighborhoods in the entire United States it wouldn’t merit targeted attention under the guise of environmental justice [31]. Alternatively, the most critical areas to focus on in terms of environmental justice are not necessarily those with the absolute lowest levels of income among its residents, which is the most common output when using tools such as EJSCREEN.

Rather than focusing solely on absolute pollution levels or relative income as a measure of potential increased vulnerability, an alternative approach in thinking about environmental justice for outdoor air pollution is to consider the trade-offs that occur in deciding where to live within a city. From this perspective, the areas of greatest concern in regards to environmental justice for air pollution would correspond to locations that must endure the adverse impacts of living in proximity to a dense urban core (i.e., elevated levels of outdoor air pollution) without experiencing the primary benefits of city living (i.e., high levels of job accessibility). While there is room to debate whether the positive aspects of urban living can offset the negative aspects of air pollution, it is less controversial to conclude that having low access to jobs while experiencing disproportionately high levels of air pollution is inequitable. 

The greater the imbalance of these trade-offs, the greater the cause for concern in regards to environmental justice. This has been termed “relative distribution inequality”, in which those receiving the benefits of capitalist production are less burdened by the resulting industrial pollution impacts than those not receiving these benefits. An examination of recent environmental inequality studies, defined by race and income, has found strong evidence for this phenomenon [32]. However, it is also important to test a wide variety of indicators, since a single definition limits the possible conclusions and resulting policy options. This recommendation is employed in the present study, with the inclusion of job accessibility as a new indicator towards refining the definition of environmental justice. Other novel indicators are possible too; for example, one study looked beyond the traditional environmental justice indicators of race and poverty by examining the relationship between household vehicle ownership and environmental exposure and health risk. Results revealed that households without vehicles were more likely to live in places with the highest pollution exposures and health burdens [33]. Different definitions of environmental justice will result in different conclusions [32]; thus, it is critical to examine various indicators in order to fully understand environmental inequities.

By using real world data for Allegheny County, PA, this study demonstrated how this improved approach in thinking about environmental justice for air pollution could be used to identify priority areas of concern within a local jurisdiction. In comparison to areas identified using traditional indicators such as those used in EJSCREEN, the inclusion of job accessibility as an environmental justice indicator resulted in the identification of areas with not only much greater levels of ambient pollution but also with dramatically lower access to jobs.

The exact configurations of the environmental justice indicator demonstrated in this study is not intended to be precisely followed in future analysis. In fact, many of the criteria selected in this analysis could be readily modified in future applications. Instead of prescribing the exact configuration of parameters to use when calculating job accessibility or the precise cut-off points to use in identifying primary and secondary areas of concern, the method outlined here is meant to serve as an illustrative example of how a new environmental indicator—job accessibility—could be incorporated into current screening methods in order to identify areas suffering from the greatest environmental inequities. For instance, racial disparities are a well-studied and key issue surrounding environmental justice literature and efforts, and may be one of the largest demographic contributors to air pollution burden among local populations [34]. In 2017, the Allegheny County Health Department performed its own environmental justice analysis using a locally-designed index, with results suggesting that the minority statistic is an important demographic variable for determining neighborhoods with the highest environmental justice needs [35].

As such, a minority variable could have been used in the present analysis alongside job accessibility to identify priority areas for environmental justice efforts. In order to test this scenario, a sensitivity analysis was performed by replacing the income variable with minority population statistics as a cut off for this study’s priority areas for environmental justice. Instead of prioritizing areas with annual median household incomes below the county average, census block groups with minorities consisting of over 50% of the block group’s population were flagged. Summary results are shown in Table 2. Comparing these priority block groups with Table 1, all six block groups identified by incorporating a minority cut off were already included in the analysis without a minority variable. Furthermore, the study analysis results in Table 1 capture a larger number of minority individuals compared to the sensitivity analysis (11,221 versus 3902, respectively). This suggests that income-focused criteria may actually capture a larger portion of minority individuals, even if these individuals don’t live in minority-majority neighborhoods. Additionally, results in Table 1 show that while the EJSCREEN analysis captures a larger number of minority individuals (15,921 versus 6164 for primary EJSCREEN and current study primary priority areas, respectively), those captured in the present analysis suffer greater environmental inequities. Specifically, EJSCREEN primary priority areas show relatively low PM_2.5_ levels (11 µg/m^3^) along with high job accessibility (436,933 jobs in a 45 min commute), while the present analysis targets areas with significantly higher pollution levels (13.8 µg/m^3^) and very low job accessibility (28,729 jobs in a 45 min commute). Thus, though the current analysis targets fewer minority individuals overall, the minority residents living in neighborhoods flagged experience much greater environmental inequities and would benefit most from targeted interventions.

Job accessibility has been measured in many ways in past research, including job density and distance from home locations [36]. In the present analysis job accessibility was measured through a mobility index defined as the average number of jobs available within a specified commute time [37]. This method allows measurements to cover various transportation methods, and provides estimates that are more realistic than simple measures of density or distance—which are meaningless if individuals have poor transportation options. In fact, it is typically low-income populations that have the lowest mobility levels and endure the longest commute times, which can result in a wide array of health and social burdens [38,39,40]. Improving mobility for disadvantaged populations thus has immense potential to improve their health and wellbeing.

Previous studies have calculated mobility index values for the Pittsburgh region (although none have incorporated job accessibility as a parameter in an environmental justice indicator). A similar calculation of job accessibility was performed in a report from the Metropolitan Policy Program at Brookings Institution by Tomer and colleagues, estimating that approximately 8.1% of available jobs in Pittsburg, PA are accessible within a 45 min public transit commute for each block group [41]. In this study, this number was closer to 25%. These discrepancies can be traced to variations in how job accessibility was calculated, including differences in travel speeds and distances. More specifically, the Tomer and colleagues study focused solely on morning commute times, while the current analysis averaged three separate daily travel times which resulted in higher job accessibility. The present analysis also included biking as a travel option which increases the number of jobs available within a 45 min commute. In regards to using job accessibility as part of an environmental justice indicator, the most important aspect is not the precise specifications of the variables in generating estimates of job accessibility but ensuring that it is used in a way that allows for the identification of locations with relatively low access to jobs within a given jurisdiction.

In the current study, travel time for commuting was limited to 45 min to calculate job accessibility, even though information from the U.S. Census Bureau indicates that just over 60% of Allegheny County public transit users commute within this timeframe [42]. Increasing commute time in this analysis to 60 min would raise the proportion of public transit users covered to 80%. While future analysis may want to conduct sensitivity analysis using longer commute times to cover a larger proportion of the population, a slightly shorter travel period was selected for use in this study to highlight the relationship between long commute times and poverty. Specifically, the average worker using public transit in Allegheny County makes significantly less annually than those who drive ($30,223 versus $41,045, respectively), and average public transit users experience longer commute times than those who drive (39 min versus 26 min, respectively) [42]. The increased travel time of lower income populations, including so called “extreme commuting”, is increasingly becoming the face of urban poverty [43].

While the purpose of any environmental justice indicator is to identify areas which experience disproportionate burdens of air pollution, the realized benefit of any such exercise only comes from the implementation of actions that seek to address the problem. Unfortunately, the spatial factors that result in elevated pollution concentrations within a city are typically non-modifiable (e.g., proximity to roadways and geographic features), so targeted mitigation of outdoor pollution levels is often not possible short of general efforts to reduce emissions and prevent the expansion of new emission sources within a city. However, even if reducing the levels of outdoor pollution is not possible, other management options to reduce the burdens associated with increased pollution levels are available, such as providing financial support for improved home insulation, provision of in-home filtration products, or prioritization of hyper-local emission reduction strategies such as conversion of school buses to cleaner fuels [44,45,46,47].

An added advantage of the inclusion of job accessibility as a parameter in calculating an air pollution environmental justice indicator is the wider range of management options that can be used to ameliorate the problem once target areas have been identified. In addition to efforts to reduce the negative aspects of elevated air pollution within a city (which may not be possible in some circumstances), it is also possible to consider efforts to improve the positive aspects of living in proximity to an urban center as a way to reduce environmental justice disparities within a city. Low-income, minority, and other traditionally underserved populations typically rely on public transit options, often traveling over twice as long during their daily commutes compared to vehicle owners [48]. Long commute times have been shown to significantly increase exposure to outdoor air pollution, increasing the risk for pollution-related health impacts [49,50]. If public transit is poorly funded and underdeveloped, commute times will be longer and fewer accessible jobs will be available to susceptible populations relying on this form of transportation. Efforts to improve public transportation systems in order to increase access to jobs, including expanded or modified service, reduced transfer times, and other efforts to reduce door-to-door commute times, may be even more important than reducing ambient pollution levels in addressing environmental disparities within cities [48,51].

There are limitations in data availability when using job accessibility as a parameter in an air pollution environmental justice indicator. Specifically, the current analysis combined spatially continuous estimates of both ambient air pollution and job accessibility in order to identify priority areas experiencing disproportionate impacts of outdoor air pollution. While estimating job accessibility through the use of mobility indices is fairly straightforward for most U.S. cities using publically available databases, the calculation of spatially continuous air pollution estimates is less likely to be available for some urban areas. Alternatives to using estimates from a local land-use regression model include available national pollution datasets such as the Downscaler Model from the U.S. EPA [52].

## 5. Conclusions

Environmental justice has made great strides in recent decades. In doing so, its definition has evolved and expanded, and it is time for the relevant policy analysis to follow. Acknowledging this need, a refined method for identifying environmental justice concerns has been presented at the local level. Inclusion of a job accessibility indicator ensures that those target areas are truly places where environmental justice is a concern. By defining environmental justice priority areas as areas with both relatively high pollution and low job accessibility, efforts can be directed to those experiencing the worst of urban living. Ultimately, incorporating employment opportunities into environmental justice analysis acknowledges personal empowerment in residential choices, while better identifying those areas where populations suffer from the negative aspects of cities without the benefits.

## Figures and Tables

**Table 1:** Summary of primary and secondary census block groups identified as environmental justice priority areas by EJSCREEN and the present study.

				Population-Weighted Averages (Percentiles Relative to County Data in Parenthesis)		
Environmental Justice Indicator	Census Block Groups	Total Population	Minority Population	Annual PM_2.5_ in µg/m^3^	Median Annual Household Income	Jobs Accessible ^a^
EJSCREEN Primary Areas ^b^	14	18,596	15,921 (86%)	11.0 (63)	$21,016 (10)	436,933 (66)
EJSCREEN Secondary Areas ^c^	37	39,921	28,906 (72%)	11.0 (62)	$23,896 (12)	493,061 (73)
Present Study Primary Areas ^d^	43	38,693	6164 (16%)	13.8 (94)	$36,941 (26)	28,720 (17)
Present Study Secondary Areas ^e^	16	13,803	5057 (37%)	13.6 (93)	$30,098 (18)	64,047 (28)

^a^ Includes jobs accessible within a 45 min commute via public transit, biking, and/or walking; ^b^ Includes block groups with 95th percentile and above EJSCREEN index values; ^c^ Includes block groups with 90th to 95th percentile EJSCREEN index values; ^d^ Includes block groups with PM_2.5_ > 12 µg/m^3^, income < $54,357, and job accessibility < 50,000; ^e^ Includes block groups with PM_2.5_ > 12 µg/m^3^, income < $54,357, and job accessibility 50,000–100,000.

**Table 2:** Summary of primary and secondary census block groups identified as environmental justice priority areas by sensitivity analysis for a minority variable cut off.

				Population-Weighted Averages (Percentiles Relative to County Data in Parenthesis)		
Environmental Justice Indicator	Census Block Groups	Total Population	Minority Population	Annual PM_2.5_ in µg/m^3^	Median Annual Household Income	Jobs Accessible ^a^
Sensitivity Analysis Primary Areas ^b^	1	1916	1038 (54%)	13.0 (100)	$49,063 (100)	32,827 (100)
Sensitivity Analysis Secondary Areas ^c^	5	3991	2864 (72%)	13.6 (92)	$16,686 (78)	73,241 (12)

*Note.* All six census block groups identified as priority areas in the sensitivity analysis using minority as a cutoff parameter were already identified as priority areas by the present study in Table 1. See Tables S3 and S4 for a direct comparison, which indicate crossover block groups with an asterisk (*). ^a^ Includes jobs accessible within a 45 min commute via public transit, biking, and/or walking; ^b^ Includes block groups with PM_2.5_ > 12 µg/m^3^, minority population > 50%, and job accessibility < 50,000; ^c^ Includes block groups with PM_2.5_ > 12 µg/m^3^, minority population > 50%, and job accessibility 50,000–100,000.

## Supplementary Materials

The following are available online at http://www.mdpi.com/1660-4601/15/6/1260/s1, Table S1: Primary block groups identified by EJSCREEN as environmental justice priority areas, Table S2: Secondary block groups identified by EJSCREEN as environmental justice priority areas, Table S3: Primary block groups identified in the present study as environmental justice priority areas, Table S4: Secondary block groups identified in the present study as environmental justice priority areas.

## References

[1] Konisky D.M. (2015). Failed Promises: Evaluating the Federal Government’s Response to Environmental Justice.

[2] Bullard R.D. (2015). Environmental justice in the United States. International Encyclopedia of the Social & Behavioral Sciences.

[3] Bullard R.D., Mohai P., Saha R., Wright B. (2007). Toxic Wastes and Race at Twenty 1987–2007.

[4] Ard K. (2015). Trends in Exposure to Industrial Air Toxins for Different Racial and Socioeconomic Groups: A Spatial and Temporal Examination of Environmental Inequality in the U.S. from 1995 to 2004. Soc. Sci. Res..

[5] United States Environmental Protection Agency (U.S. EPA) (2018). Environmental Justice 2017 Progress Report.

[6] U.S. EPA (2017). EJSCREEN Technical Documentation.

[7] U.S. EPA (2015). Guidance on Considering Environmental Justice during the Development of Regulatory Actions.

[8] Rosofsky A., Levy J.I., Zanobetti A., Janulewicz P., Fabian M.P. (2018). Temporal Trends in Air Pollution Exposure Inequality in Massachusetts. Environ. Res..

[9] Angel S., Blei A.M., Parent J., Lamson-Hall P., Sánchez N.G. (2016). Atlas of Urban Expansion—2016 Edition, Volume 1: Areas and Densities.

[10] Bertaud A. (2014). Cities as Labor Markets.

[11] Bertaud A. (2004). The Spatial Organization of Cities: Deliberate Outcome or Unforeseen Consequence?.

[12] Fullerton A.S., Villemez W.J. (2011). Why Does the Spatial Agglomeration of Firms Benefit Workers? Examining the Role Oforganizational Diversity in U.S. Industries and Labor Markets. Soc. Forces.

[13] Vozar S., Holt A. (2017). 2016 Air Quality Annual Report.

[14] U.S. EPA PM-2.5 Nonattainment Areas by State/County/Area. https://www3.epa.gov/airquality/greenbook/kncty.html.

[15] Li H.Z., Dallmann T.R., Gu P., Presto A.A. (2016). Application of Mobile Sampling to Investigate Spatial Variation in Fine Particle Composition. Atmos. Environ..

[16] Eeftens M., Beelen R., de Hoogh K., Bellander T., Cesaroni G., Cirach M., Declercq C., Dedele A., Dons E., de Nazelle A. (2012). Development of Land Use Regression Models for PM(2.5), PM(2.5) Absorbance, PM and PM(Coarse) in 20 European Study Areas; Results of the Escape Project. Environ. Sci. Technol..

[17] OpenTripPlanner Version 1.2. www.opentripplanner.org.

[18] Busby J.R. (2004). Accessibility-Based Transit Planning.

[19] Ducas C.R. (2011). Incorporating Livability Benefits into the Federal Transit Administration New Starts Project Evaluation Process through Accessibility-Based Modeling.

[20] Warade R.K. (2007). The Accessibility and Development Impacts of New Transit Infrastructure: The Circle Line in Chicago.

[21] Owen A., Murphy B., Levinson D.M. (2016). Access across America: Transit 2015: Accessibility Observatory. Center for Transportation Studies.

[22] Python Language Reference Version 3.6. https://docs.python.org/3/download.html.

[23] LEHD Origin-Destination Employment Statistics (2002–2015) Version LODES 7.3.

[24] U.S. Census Bureau (2017). 2012–2016 American Community Survey 5-Year Estimates.

[25] Folch D.C., Arribas-Bel D., Koschinsky J., Spielman S.E. (2016). Spatial Variation in the Quality of American Community Survey Estimates. Demography.

[26] Moretti E. (2010). Local Multipliers. Am. Econ. Rev..

[27] Moretti E., Thulin P. (2013). Local Multipliers and Human Capital in the United States and Sweden. Ind. Corp. Chang..

[28] Van Dijk J.J. (2016). Local Employment Multipliers in U.S. Cities. J. Econ. Geogr..

[29] Anderson R.R., Martello D.V., White C.M., Crist C.C., John K., Modey W.K., Eatough D.J. (2012). The Regional Nature of PM2.5 Episodes in the Upper Ohio River Valley. J. Air Waste Manag. Assoc..

[30] Tang W., Raymond T., Wittig B., Davidson C., Pandis S., Robinson A., Crist K. (2004). Spatial Variations of PM2.5 during the Pittsburgh Air Quality Study. Aerosol. Sci. Technol..

[31] New York City Department of Health and Mental Hygiene (2018). The New York City Community Air Survey: Neighborhood Air Quality 2008–2016.

[32] Downey L. (2005). Assessing Environmental Inequality: How the Conclusions We Draw Vary According to the Definitions We Employ. Sociol. Spectr..

[33] Chakraborty J. (2009). Automobiles, Air Toxics, and Adverse Health Risks: Environmental Inequities in Tampa Bay, Florida. Ann. Assoc. Am. Geogr..

[34] Lievanos R.S. (2018). Retooling Calenviroscreen: Cumulative Pollution Burden and Race-Based Environmental Health Vulnerabilities in California. Int. J. Environ. Res. Public Health.

[35] Lewis J., Huerbin T., Brink L. (2017). Allegheny County Environmental Justice: Identifying High Priority Communities in Allegheny County.

[36] Geurs K.T., van Wee B. (2004). Accessibility Evaluation of Land-Use and Transport Strategies: Review and Research Directions. J. Transp. Geogr..

[37] Bertaud A. (2016). Mobility Transport Is a Real Estate Issue—The Design of Urban Roads and Transport Systems.

[38] Hansson E., Mattisson K., Bjork J., Ostergren P.O., Jakobsson K. (2011). Relationship between Commuting and Health Outcomes in a Cross-Sectional Population Survey in Southern Sweden. BMC Public Health.

[39] Hoehner C.M., Barlow C.E., Allen P., Schootman M. (2012). Commuting Distance, Cardiorespiratory Fitness, and Metabolic Risk. Am. J. Prev. Med..

[40] Mattisson K., Håkansson C., Jakobsson K. (2015). Relationships between Commuting and Social Capital among Men and Women in Southern Sweden. Environ. Behav..

[41] Tomer A., Kneebone E., Puentes R., Berube A. (2011). Missed Opportunity: Transit and Jobs in Metropolitan America.

[42] U.S. Census Bureau 2012–2016 American Community Survey 5-Year Estimates: Means of Transportation to Work by Selected Characteristics. https://factfinder.census.gov/faces/tableservices/jsf/pages/productview.xhtml?pid=ACS_16_5YR_S0802&prodType=table.

[43] Bertaud A. (2012). Converting Land into Affordable Housing Floor Space. World Bank’s Sixth Urban Research and Knowledge Symposium.

[44] Fitz D.R., Winer A.M., Colome S. (2003). Characterizing the Range of Children’s Pollutant Exposure During School Bus Commutes.

[45] Gao O.H., Klein R.A. (2010). Environmental Equity in Participation of the Clean Air School Bus Program: The Case of New York State. Transp. Res. D.

[46] Environmental Law Institute (2016). Addressing Indoor Air Quality in School Energy Efficiency Upgrades: Review of Selected State Policies.

[47] Shimer D., Phillips T.J., Jenkins P.L. (2005). Report to the California Legislature: Indoor Air Pollution in California.

[48] Epting S. (2016). A Different Trolley Problem: The Limits of Environmental Justice and the Promise of Complex Moral Assessments for Transportation Infrastructure. Sci. Eng. Ethics.

[49] Ragettli M.S., Phuleria H.C., Tsai M.Y., Schindler C., de Nazelle A., Ducret-Stich R.E., Ineichen A., Perez L., Braun-Fahrlander C., Probst-Hensch N. (2015). The Relevance of Commuter and Work/School Exposure in an Epidemiological Study on Traffic-Related Air Pollution. J. Expo. Sci. Environ. Epidemiol..

[50] Tang R., Tian L., Thach T.Q., Tsui T.H., Brauer M., Lee M., Allen R., Yuchi W., Lai P.C., Wong P. (2018). Integrating Travel Behavior with Land Use Regression to Estimate Dynamic Air Pollution Exposure in Hong Kong. Environ. Int..

[51] U.S. Department of Transportation Federal Highway Administration (2015). Federal Highway Administration Environmental Justice Reference Guide.

[52] U.S. EPA Downscaler Model for Predicting Daily Air Pollution. https://www.epa.gov/air-research/downscaler-model-predicting-daily-air-pollution.

