# Supplementary material for: A Novel Environmental Justice Indicator for Managing Local Air Pollution
Source: Int J Environ Res Public Health. 2018 Jun 14;15(6):1260. doi: 10.3390/ijerph15061260 (PMC6024918; doi:10.3390/ijerph15061260)
Supplement: Supplementary file 1 [file ijerph-15-01260-s001.pdf]

Supplementary Materials

A Novel Environmental Justice Indicator for Managing Local Air Pollution

Jing Zhao, Laura Gladson and Kevin Cromar *

Marron Institute of Urban Management, New York University, 60 5th Avenue, New York, NY 10011, USA; jz2181@nyu.edu (J.Z.); laura.gladson@nyu.edu (L.G.)

* Correspondence: kevin.cromar@nyu.edu; Tel.: +1-212-992-6839

Table S1. Primary block groups identified by EJSCREEN as environmental justice priority areas. 14 block groups were identified by traditional analysis as primary environmental justice concerns, defined as those with EJSCREEN $PM_{2.5}$ environmental index values at or above the 95th percentile level.

**Census Block Group** (Tract + Block FIPS)	**Population**	**Minority Population**	**Annual $PM_{2.5}$** ($\mu g/m^3$)	**Income** (median annual household)	**Jobs Accessible**[a]
030500 1	1,299	1,155	10.9	$19,914	970,175
050900 1	1,367	1,367	11.1	$14,281	822,029
130100 1	822	822	9.8	$21,208	554,949
130600 3	1,623	1,509	9.9	$13,871	512,867
260900 1	1,440	1,403	9.8	$12,742	454,460
271500 2	1,038	645	10.5	$34,048	514,558
300100 4	1,501	1,134	9.9	$21,755	541,024
504100 4	1,554	1,259	11.0	$23,958	55,865
512800 1	1,478	979	14.8	$19,517	290,229
514000 2	1,480	1,343	16.4	$20,761	498,185
551200 1	1,114	781	9.9	$9,458	74,741
561400 3	1,299	1,272	9.8	$31,832	580,427
561900 2	1,445	1,311	9.8	$31,745	147,315
561900 1	1,136	941	9.8	$21,637	131,062
**Total**	**18,596**	**15,921**	**-**	**-**	**-**
**Population Weighted Averages**	**-**	**-**	**11.0**	**$21,016**	**436,933**

a. Includes jobs accessible within a 45 minute commute via public transit, biking, and/or walking.

Table S2. Secondary block groups identified by EJSCREEN as environmental justice priority areas. A total of 37 block groups were identified by traditional analysis as secondary environmental justice concerns, defined as those with EJSCREEN $PM_{2.5}$ environmental index values between the 90th and 95th percentile levels.

**Census Block Group** (Tract + Block FIPS)	**Population**	**Minority Population**	**Annual $PM_{2.5}$** ($\mu g/m^3$)	**Income** (median annual household)	**Jobs Accessible**[a]
030500 2	993	967	10.2	$17,673	954,187
040200 1	888	730	10.5	$13,229	825,088
040500 2	2,120	375	9.9	$16,806	786,905
040600 1	1,938	332	11.3	$19,659	771,036
051000 1	913	681	10.4	$15,929	806,621
101600 1	1,071	1,034	9.8	$27,019	637,124
101700 2	871	623	9.8	$33,906	693,053
111400 2	892	777	9.8	$22,000	685,394
111500 4	693	682	11.6	$21,326	723,676
111500 2	1,500	972	11.1	$28,443	769,081
120300 2	582	582	11.2	$22,206	565,730
120300 1	1,079	1,041	11.7	$35,000	562,508
130100 2	591	591	10.0	$21,538	608,020
130200 2	867	821	10.2	$36,447	677,051
261400 3	559	454	9.8	$15,556	563,715
281400 2	1,706	1,118	10.1	$25,417	632,618
300100 2	651	618	9.9	N/A	559,477
477300 1	1,252	872	9.8	$29,250	175,412
480101 3	1,056	547	13.1	$32,119	239,721
483800 2	1,288	974	12.6	$19,063	441,160
483800 3	1,189	876	10.1	$25,662	355,997
486700 1	1,549	1,049	12.2	$16,772	270,944
492800 1	1,255	856	11.9	$26,555	55,391
513800 2	784	569	14.5	$19,922	335,083
523100 1	1,387	1,246	10.1	$45,284	211,904
551900 2	1,061	688	13.8	$12,176	94,982
552100 2	1,000	708	13.4	$14,097	75,451
552300 3	1,227	703	10.8	$22,204	68,907
560400 2	1,077	771	11.3	$31,319	652,145
561000 1	766	749	9.8	$23,750	651,597
561100 1	867	832	9.8	$20,262	636,755
561400 4	1,419	1,269	9.9	$31,435	520,064
561500 3	843	762	9.8	$24,441	631,141
561500 2	913	697	9.8	$25,573	581,727
562300 2	888	709	12.4	N/A	177,726
562300 3	699	497	10.9	$25,347	180,488
562500 4	1,487	1,134	11.9	$40,625	202,551
**Total**	**39,921**	**28,906**	**-**	**-**	**-**
**Population Weighted Averages**	**-**	**-**	**11.0**	**$23,896**	**493,061**

a. Includes jobs accessible within a 45 minute commute via public transit, biking, and/or walking.

Table S3. Primary block groups identified in the present study as environmental justice priority areas. A total of 43 block groups were identified in the present study as primary environmental justice concerns, defined as those with $PM_{2.5}$ >12 μg/m^3, income < $54,357, and job accessibility < 50,000.

**Census Block Group** (Tract + Block FIPS)	**Population**	**Minority Population**	**Annual $PM_{2.5}$** (μg/m^3)	**Income** (median annual household)	**Jobs Accessible**[a]
401100 2	1,400	67	12.8	$54,096	8,117
401200 1	564	87	16.5	$18,510	20,068
401200 3	764	57	16.5	$31,250	20,374
401200 2	751	18	14.7	$15,813	21,034
401200 4	888	0	14.9	$42,114	27,722
402000 3	1,248	141	12.0	$49,141	30,303
402000 4	661	54	13.9	$23,500	29,130
402000 2	663	2	14.9	$43,047	29,667
403500 4	1,052	254	12.4	$20,744	29,953
403500 5	745	93	12.8	$27,841	29,083
403500 3	908	90	12.1	$31,320	30,430
416000 1	910	7	13.2	$44,167	32,479
417100 1	842	39	13.3	$53,864	33,064
417200 1	1,319	1	14.6	$34,055	33,398
419000 2	710	46	14.6	$45,391	44,805
419000 1	1,219	60	15.4	$37,256	36,226
450800 2	374	170	12.2	$36,429	42,179
450800 1	1,580	646	12.0	$28,654	25,184
492700 2	680	274	15.6	$43,958	44,058
492700 3	1,008	216	15.5	$35,625	36,825
492700 1	341	34	14.2	N/A	41,578
492900 2	492	244	13.6	$39,107	44,994
494000 1	1,437	82	12.2	$37,014	20,322
498000 2	1,363	111	12.5	$53,750	21,694
498000 1	1,191	69	12.1	$50,104	18,125
499300 1	551	17	15.6	$43,125	47,956
499400 2	738	52	13.3	$29,207	39,761
499400 3	1,047	73	15.9	$36,400	41,356
501000 1	1,650	132	12.9	$30,324	27,379
523501 1*	1,916	1,038	13.0	$49,063	32,827
523502 1	1,479	418	13.8	$33,056	41,746
523702 4	544	233	13.1	$42,948	48,386
524000 1	780	118	12.4	$50,064	31,387
524000 3	752	68	12.9	$50,833	30,254
525200 1	1,526	2	14.1	$41,210	39,891
526101 1	643	45	13.9	$47,400	2,141
552400 3	1,059	129	13.4	$36,979	48,307
564200 1	434	30	12.1	$49,286	89
564500 3	515	36	15.2	$47,667	4,720
980600 1	4	0	13.5	N/A	0
980700 1	14	0	16.9	N/A	0

Census Block Group (Tract + Block FIPS)	Population	Minority Population	Annual $PM_{2.5}$ (μg/m^3)	Income (median annual household)	Jobs Accessible[a]
980800 1	N/A	N/A	13.8	N/A	0
980900 1	1,931	911	18.1	N/A	0
**Total**	**38,693**	**6,164**	**-**	**-**	**-**
**Population Weighted Averages**	**-**	**-**	**13.8**	**$36,941**	**28,720**

a. Includes jobs accessible within a 45 minute commute via public transit, biking, and/or walking.
* Census block group also identified as a primary priority area for the sensitivity analysis summarized in Table 2.

Table S4. Secondary block groups identified in the present study as environmental justice priority areas. A total of 16 block groups were identified in the present study as secondary environmental justice concerns, defined as those with $PM_{2.5}$ >12 μg/m^3, income < $54,357, and job accessibility between 50,000 and 100,000.

Census Block Group (Tract + Block FIPS)	Population	Minority Population	Annual $PM_{2.5}$ (μg/m^3)	Income (median annual household)	Jobs Accessible[a]
448000 1	1,287	296	13.7	$46,389	50,663
486900 3*	217	207	12.3	N/A	99,638
487000 1	835	54	13.3	$48,542	62,170
487000 2	906	27	14.0	$31,053	70,641
488400 3	527	13	12.5	$30,588	69,732
488600 2	830	356	19.1	$18,221	60,539
492800 2*	1,216	821	14.1	$23,138	50,055
504100 2	354	25	12.1	$41,023	71,033
508000 1	828	410	13.0	$31,042	60,451
508000 2	1,083	270	12.5	$23,142	50,403
521401 1	1,826	366	13.4	$51,573	51,841
551900 2*	1,061	688	13.8	$12,176	94,982
552100 1	490	219	12.6	$18,152	78,213
552100 2*	1,000	708	13.4	$14,097	75,451
552200 1	846	157	13.1	$24,688	71,928
552300 2*	497	440	12.8	$23,021	67,582
**Total**	**13,803**	**5,057**	**-**	**-**	**-**
**Population Weighted Averages**	**-**	**-**	**13.6**	**$30,098**	**64,047**

a. Includes jobs accessible within a 45 minute commute via public transit, biking, and/or walking.
* Census block groups also identified as a secondary priority areas for the sensitivity analysis summarized in Table 2.
